# Draft Genome Sequence of the Phyllosphere Model Bacterium *Pantoea agglomerans* 299R

**DOI:** 10.1128/genomeA.00036-13

**Published:** 2013-02-28

**Authors:** Mitja N. P. Remus-Emsermann, Eun Bae Kim, Maria L. Marco, Robin Tecon, Johan H. J. Leveau

**Affiliations:** a Department of Microbial Ecology, Netherlands Institute of Ecology (NIOO-KNAW), Wageningen, The Netherlands; b Department of Food Science and Technology, University of California, Davis, California, USA; c Department of Plant Pathology, University of California, Davis, California, USA

## Abstract

Bacteria belonging to the genus *Pantoea* are common colonizers of plant leaf surfaces. Here, we present the draft genome sequence of *Pantoea agglomerans* 299R, a phyllosphere isolate that has become a model strain for studying the ecology of plant leaf-associated bacterial commensals.

## GENOME ANNOUNCEMENT

The phyllosphere, or air-surface interface of plant leaves, provides a habitat to a large and diverse community of microorganisms, including bacteria, yeasts, and filamentous fungi (1, 2). Through interactions with each other, their host, and the atmosphere, phyllosphere microorganisms impact the health of plants, humans, and the planet, for example as foliar pathogens or disease protectants of agricultural crops (3), as enteropathogenic contaminants on fresh produce (4), or as ice-nucleating agents contributing to cloud formation (5). Exposure to UV radiation and desiccation renders the phyllosphere a hostile environment that demands great functional hardiness and plasticity from its microbial inhabitants (6).

*Pantoea agglomerans* 299R (*Pa*299R; syn. *Erwinia herbicola* 299R) is a spontaneous rifampin-resistant derivative of isolate 299, a pigmented bacterium that was recovered from healthy leaves of a Bartlett pear tree near Healdsburg, CA (7). As a model organism for the study of nonpathogenic bacterial epiphytes, *Pa*299R has contributed greatly to our understanding of phyllosphere-specific adaptations (8), patterns of bacterial aggregation and dispersion on leaves (9, 10), competition for space and nutrients (11, 12), lateral heterogeneity of the phyllosphere environment (13, 14), and impact of this heterogeneity on bacterial survival and growth (15, 16). Availability of the *Pa*299R genome sequence will facilitate “omics”-based studies with this model strain and accelerate the discovery of bacterial genes underlying phyllosphere fitness.

*Pa*299R was obtained from Steve Lindow (University of California, Berkeley). Genomic DNA was isolated from an overnight Luria-Bertani (LB) culture using a DNeasy blood and tissue kit (Qiagen, Venlo, The Netherlands) and paired-end Illumina-sequenced by BaseClear (Leiden, The Netherlands). Total RNA was isolated using RNA Protect and an RNeasy Mini Kit (Qiagen) from cultures on 0.4% glucose M9 medium with or without 0.2% Casamino Acids and sent to the UC Davis Genome Center for rRNA depletion using a Ribo-zero rRNA removal kit (Epicentre, Madison, WI), library construction, and Illumina HiSeq2000 sequencing (single reads, 50 cycles). After quality filtering, 583 Mbp of DNA and 651 Mbp of unique RNA reads were assembled, using Ray 1.7 (17) and a 21-bp kmer size, into 109 contigs (≥200 bp, N_50_ length: 109,356 bp; average length, 42,031 bp), representing 4,581,483 bp of genomic DNA (269-fold coverage) with a G+C content of 54.29%. Gene prediction by RAST (18) uncovered 4,194 coding sequences and 62 tRNAs.

The *Pa*299R genome revealed many adaptations consistent with an epiphytic lifestyle, including genes for high-affinity uptake and utilization of the photosynthates sucrose, fructose, and glucose, for repair of UV-damaged DNA, and for production of the osmoprotectants betaine and trehalose. *Pa*299R possesses a *Pantoea*-typical LPP-1 plasmid (19), coding for the biosynthesis of thiamine and the pigment zeaxanthin. Presence of the *ipdC* gene for production of the plant hormone indole 3-acetic acid (7) was confirmed, but no genes were found for synthesis of pantocin A, a *Pantoea*-characteristic antibiotic (20). The spontaneous resistance of *Pa*299R to rifampicin resulted from a D516V substitution in the *rpoB* gene product.

### Nucleotide sequence accession numbers.

This whole-genome shotgun project has been deposited at DDBJ/EMBL/GenBank under the accession number ANKX00000000. The version described in this paper is the first version, ANKX01000000.
